# Genomic variation among closely related *Vibrio alginolyticus* strains is located on mobile genetic elements

**DOI:** 10.1186/s12864-020-6735-5

**Published:** 2020-05-11

**Authors:** Cynthia Maria Chibani, Olivia Roth, Heiko Liesegang, Carolin Charlotte Wendling

**Affiliations:** 1 Department of Genomic and Applied Microbiology, Institute of Microbiology and Genetics, Georg-August-University, 37077 Göttingen, Germany; 2 Institute for Microbiology and Genetics, Georg-August University Goettingen, Grisebachstr. 8, 37077 Goettingen, Germany; 3 GEOMAR, Helmholtz Centre for Ocean Research, Marine Evolutionary Ecology, Duesternbrooker Weg 20, 24105 Kiel, Germany; 4 Department of Environmental Systems Science, ETH Zürich, Universitätsstraße 16, 8092 Zürich, Switzerland

**Keywords:** Pangenome, Mega-plasmids, Mobile genetic elements, Horizontal gene transfer, Genomic islands, Niche-adaptation

## Abstract

**Background:**

Species of the genus *Vibrio*, one of the most diverse bacteria genera, have undergone niche adaptation followed by clonal expansion. Niche adaptation and ultimately the formation of ecotypes and speciation in this genus has been suggested to be mainly driven by horizontal gene transfer (HGT) through mobile genetic elements (MGEs). Our knowledge about the diversity and distribution of *Vibrio* MGEs is heavily biased towards human pathogens and our understanding of the distribution of core genomic signatures and accessory genes encoded on MGEs within specific *Vibrio* clades is still incomplete. We used nine different strains of the marine bacterium *Vibrio alginolyticus* isolated from pipefish in the Kiel-Fjord to perform a multiscale-comparative genomic approach that allowed us to investigate [1] those genomic signatures that characterize a habitat-specific ecotype and [2] the source of genomic variation within this ecotype.

**Results:**

We found that the nine isolates from the Kiel-Fjord have a closed-pangenome and did not differ based on core-genomic signatures. Unique genomic regions and a unique repertoire of MGEs within the Kiel-Fjord isolates suggest that the acquisition of gene-blocks by HGT played an important role in the evolution of this ecotype. Additionally, we found that ~ 90% of the genomic variation among the nine isolates is encoded on MGEs, which supports ongoing theory that accessory genes are predominately located on MGEs and shared by HGT. Lastly, we could show that these nine isolates share a unique virulence and resistance profile which clearly separates them from all other investigated *V. alginolyticus* strains and suggests that these are habitat-specific genes, required for a successful colonization of the pipefish, the niche of this ecotype.

**Conclusion:**

We conclude that all nine *V. alginolyticus* strains from the Kiel-Fjord belong to a unique ecotype, which we named the Kiel-*alginolyticus* ecotype. The low sequence variation of the core-genome in combination with the presence of MGE encoded relevant traits, as well as the presence of a suitable niche (here the pipefish), suggest, that this ecotype might have evolved from a clonal expansion following HGT driven niche-adaptation.

## Background

The genomic variation within a bacterial species is reflected by accessory genes. Together with strain-specific singletons (genes found in only one strain) and the core-genes that are shared by all strains of a species (such as housekeeping genes and genes required for essential cellular functions) the accessory elements form the pangenome [1]. Accessory genes are usually only found in a subset of genomes of a species and are attributed to habitat-specific functions, such as strain-specific virulence factors [2], antibiotic resistance [3], or niche-adaptation [4]. As such, accessory genes are often located on mobile genetic elements (MGEs), including plasmids, bacteriophages, transposons, integrative or conjugative elements (ICEs) and genomic islands (GIs) and are shared between species via horizontal gene transfer (HGT) [5]. Even though HGT between strains of the same species is more frequent, some MGEs have a broad host range enabling them to move genes across species boundaries thereby blurring the definition of a species [6].

Not only the size of pangenomes differs among species but also the relationship between the proportion of core-genes relative to the pangenome [7]. A large pangenome with a proportionally small core-genome and with high rates of HGT is considered open, whereas a small pangenome with a high proportion of core-genes and low HGT rates is considered to be closed [8]. For a wide range of bacteria, the pangenome size is positively correlated with the effective population size (N_e_) [7]. Species with a high N_e_ are likely to occupy different environmental niches where they experience a larger diversity of selection pressures, which will drive selection for increased genome diversity and ultimately lead to larger pangenomes [8]. However, niche adaptation in species with high N_e_ can also select for distinct ecotypes (i.e. ecologically and evolutionary distinct subpopulations) and ultimately initiate a speciation process with a niche-specific pangenome [8]. It remains debated when ecotypes are sufficiently diverged to be considered different species [9].

An excellent model organism to study pan-core genomic signatures and niche adaptation resulting from HGT is the genus *Vibrio*. These bacteria constitute one of the most diverse bacterial genera with a large pangenome comprising more than 100 species from different clades, that occupy different ecological niches [10, 11], where they can exist as pathogenic, opportunistic, mutualistic or free-living forms [12]. The considerable large pangenome (> 26,500 genes) of the genus *Vibrio*, which is more than 50 times larger than the *Vibrio* specific core-genome [11], indicates a large reservoir of genomic diversity for this genus. In contrast, within a specific *Vibrio* clade, the pangenome to core-genome ratio decreases considerably. For instance, within the *cholerae* clade, the pangenome is only 4.5 times larger than the core-genome [11]. The relatively small *cholerae*-pangenome (6923 genes) compared to the entire *Vibrio*-pangenome, might reflect a specific set of genes, required to occupy the cholera-specific niche.

Niche adaptation and ultimately speciation in this genus has been suggested to be mainly driven by HGT [13]. Furthermore, MGEs, in particular filamentous phages, contribute significantly to the emergence of pathogenic *vibrios* from environmental populations [6] and the prophage repertoire of a given *Vibrio* species can account for a large fraction of the variation among strains [14, 15]. Our knowledge about the diversity and distribution of *Vibrio* MGEs is heavily biased towards human pathogens, e.g. *V. cholerae* and *V. parahaemolyticus*. While recent studies started to expand our knowledge on *Vibrio*-specific MGEs to other clades, such as *V. anguillarum* [16, 17], *V. fischerii* [18], or *V. alginolyticus* [19], our understanding about the distribution of core-genomic signatures and accessory genes encoded on MGEs within specific *Vibrio* clades is still incomplete. This might be because most of the available genomes are draft genomes comprising multiple contigs, whereas only a few complete genomes of environmental *vibrios* are available to date [19]. It is challenging to generate high-quality genomes of *vibrios* due to repetitive genomic regions such as rRNA operons present in the two chromosomes [8–12] and arrays of multiple integrated prophages. Especially multiple prophage arrays cannot be resolved using draft genome assemblies from short-read sequencing techniques. Due to the incomplete information concerning the genome organization stored in draft genomes, it is not possible to study genome dynamics of mobile genetic elements especially of prophages without long-read sequencing data [20].

The present study aimed to investigate how genome organization, as well as core-genomic signatures and accessory genes, differ within a group of closely related environmental *Vibrio* isolates. To do so, we performed a multi-scale comparative genomics approach using *Vibrio alginolyticus* as a model organism. *V. alginolyticus,* a ubiquitous marine opportunistic pathogen can cause mass mortalities in shellfish, shrimp, and fish, resulting in severe economic losses worldwide [3, 21, 22]. *Vibrio* pathogenicity is a complex interaction of abiotic and biotic factors, including high temperatures [23, 24] low salinities [25], host and bacterial genotypes [26, 27]. To date only six closed *alginolyticus* genomes are available, while 28 remain fragmented with 2 to 75 contigs (last accessed: December 2019). A detailed comparative genomic characterization of the *alginolyticus* species, including plasmid content, distribution of MGEs and virulence factors of this species is as yet missing. Here we analysed core-and accessory-genomic signatures of *V. alginolyticus* by comparing nine closely related *V. alginolyticus* genomes, isolated from the pipefish *Syngnathus typhle* in the Kiel-Fjord [28], (later named Kiel-*alginolyticus* ecotype) with less closely related *V. alginolyticus* isolates (Table 1).

**Table 1:** All *V. alginolyticus* strains used in the present study (* circular phage replicon)

	strain	GC%	Replicon	Size [bp]	CDS	Ref	Genbank
Kiel-Fjord Vibrio alginolyticus genomes	K01M1	44.6	Chromosome 1	3,468,303	3206	This study	CP017889.1
			Chromosome 2	1,883,748	1668	This study	CP017890.1
			pL9064	9064	8	This study	CP028135.1
	K04M1	44.31	chromosome 1	3,473,127	3213	This study	CP017891.1
			chromosome 2	1,870,775	1660	This study	CP017892.1
			pL19	19,690	28	This study	CP017893.1
			pL280	280,614	305	This study	CP017894.1
			vK04M1*	7079	11	This study	CP017895.1
	K04M3	44.31	chromosome 1	3,476,174	3219	This study	CP017896.1
			chromosome 2	1,903,830	1708	This study	CP017897.1
			pL294	294,086	325	This study	CP017898.1
	K04M5	44.31	chromosome 1	3,470,916	3211	This study	CP017899.1
			chromosome 2	1,900,618	1688	This study	CP017900.1
			pL294	294,721	320	This study	CP017901.1
	K05K4	44.34	chromosome 1	3,473,579	3218	This study	CP017902.1
			chromosome 2	1,875,554	1670	This study	CP017903.1
			pL289	289,065	315	This study	CP017904.1
			vK05K4_1*	21,012	34	This study	CP017905.1
			vK05K4_2*	13,327	23	This study	CP017906.1
	K06K5	44.31	Chromosome 1	3,471,297	3213	This study	CP017907.1
			Chromosome 2	1,879,729	1662	This study	CP017908.1
			pL29	29,688	20	This study	CP017909.1
			pL291	291,285	322	This study	CP017910.1
	K08M3	44.32	Chromosome 1	3,468,139	3214	This study	CP017913.1
			Chromosome 2	1,886,577	1675	This study	CP017914.1
			pL300	300,425	331	This study	CP017915.1
	K09K1	44.61	Chromosome 1	1,897,210	3209	This study	CP017918.1
			Chromosome 2	3,465,619	1704	This study	CP017919.1
	K10K4	44.6	Chromosome 1	3,494,647	3231	This study	CP017911.1
			Chromosome 2	1,894,531	1682	This study	CP017912.1
all other closed V. alginolyticus genomes	ATCC 33787	44.48	Chromosome 1	3,362,673	3190	Wang et al. 2016 [29]	CP013484.1
			Chromosome 2	1,851,538	1674	Wang et al. 2016 [29]	CP013485.1
			pMBL128	128,112	144	Wang et al. 2016 [29]	CP013486.1
			pMBL287	286,750	301	Wang et al. 2016 [29]	CP013487.1
			pMBL96	95,866	109	Wang et al. 2016 [29]	CP013488.1
	ZJ-T	44.67	Chromosome 1	3,535,128	3301	Deng et al. 2016 [30]	CP016224.1
			Chromosome 2	1,870,966	1657	Deng et al. 2016 [30]	CP016225.1
	ATCC 17749	44.7	Chromosome 1	3,334,467	3128	Liu et al. 2015 [31]	CP006718.1
			Chromosome 2	1,812,170	1640	Liu et al. 2015 [31]	CP006719.1
	FDAARGOS_108	44.15	Chromosome 1	3,418,310	3047	Hoffmann, M (unpublished)	CP014053
			Chromosome 2	1,810,594	1587	Hoffmann, M (unpublished)	CP014054.1
			unnamed plasmid	217,123	231	Hoffmann, M (unpublished)	CP014052
	FDAARGOS_110	44.57	Chromosome 1	3,434,696	3156	Hoffmann, M (unpublished)	CP014040.1
			Chromosome 2	1,833,981	1622	Hoffmann, M (unpublished)	CP014041.1
	FDAARGOS_114	44.68	Chromosome 1	3,372,141	3054	Hoffmann, M (unpublished)	CP014045.1
			Chromosome 2	1,806,127	1593	Hoffmann, M (unpublished)	CP014044.1

## Results and discussion

### Genome features of Vibrio alginolyticus isolated from the Kiel-Fjord

We sequenced the genomes of nine *Vibrio alginolyticus* strains, previously isolated from the gut or gills of six different pipefish caught in the Kiel-Fjord (Germany, 54°75′57″N; 9°87′66″E) in May 2010 [28], using a combination of PacBio long- and Illumina short-read technology. The assembly resulted in eight closed genome sequences and one draft genome (strain K09K1), where both chromosomes have been assembled into a single contig due to multiple copies of an integrated filamentous phage. The replicon boundaries could not be resolved experimentally based on PCR thus strain K09K1 has been assigned a “permanent draft” status. All *V. alginolyticus* genomes contain a ~ 3.47 Mbp chromosome 1 and a ~ 1.88 Mbp chromosome 2, with a %GC content of around 44% (Table 1), which is typical for the genus *Vibrio* as well as for the species *V. alginolyticus* [29, 32]. We found extra-chromosomal replicons including plasmids and filamentous phages in seven isolates (Table 1).

### Species definition and phylogenetic relationship of the Kiel-Fjord isolates

**Fig. 1:**
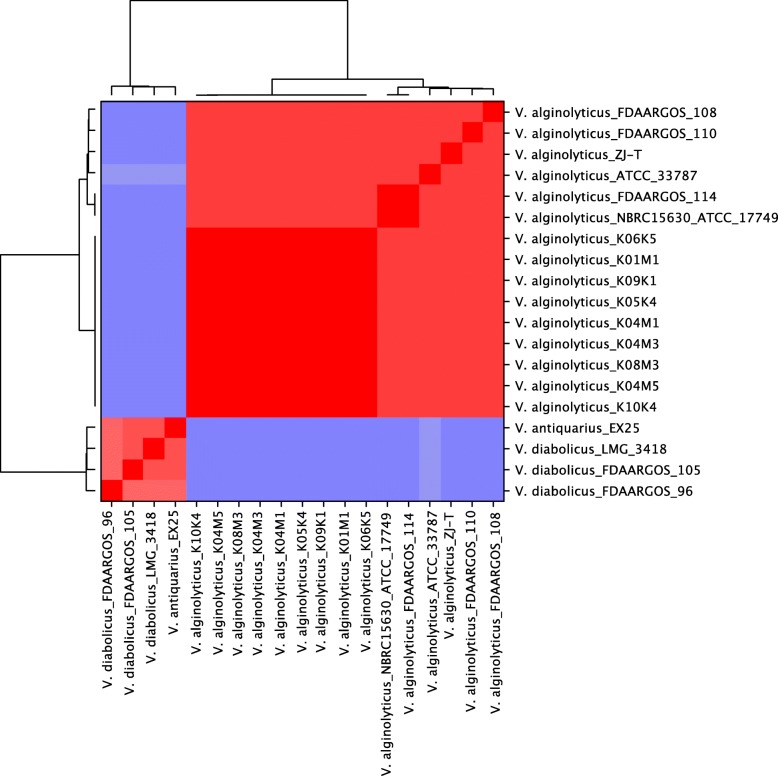
Average nucleotide identity (ANI) percentage analysis of closed *Vibrio* genomes. ANI analysis based on MUMmer alignment of the genome sequences was performed and visualized using PYANI. ANI-values ranging from 0 to 70% no hits, 70–95% ANI colored in blue and higher than 95% ANI in red) All true *V. alginolyticus* (including the nine strains from the present study) can be separated from *V. diabolicus* and *V. antiquaries* (small red square, bottom left)

All nine strains isolated from the Kiel-Fjord share more than 98% average nucleotide identity (ANI) with other *alginolyticus* strains, suggesting that these strains belong, as previously suggested based on a multi-locus-sequencing-approach (MLSA) [23], to the species *V. alginolyticus*. All *alginolyticus* strains share ~ 92% ANI with two other closely related *Vibrio* species, i.e. *V. diabolicus* and *V. antiquarius* (Fig. 1), which is below the species threshold of ANI = 95% [33, 34], indicating that *V. alginolyticus* is a distinct species.

### The Vibrio alginolyticus pan/ core genome

#### Pangenome

**Fig. 2:**
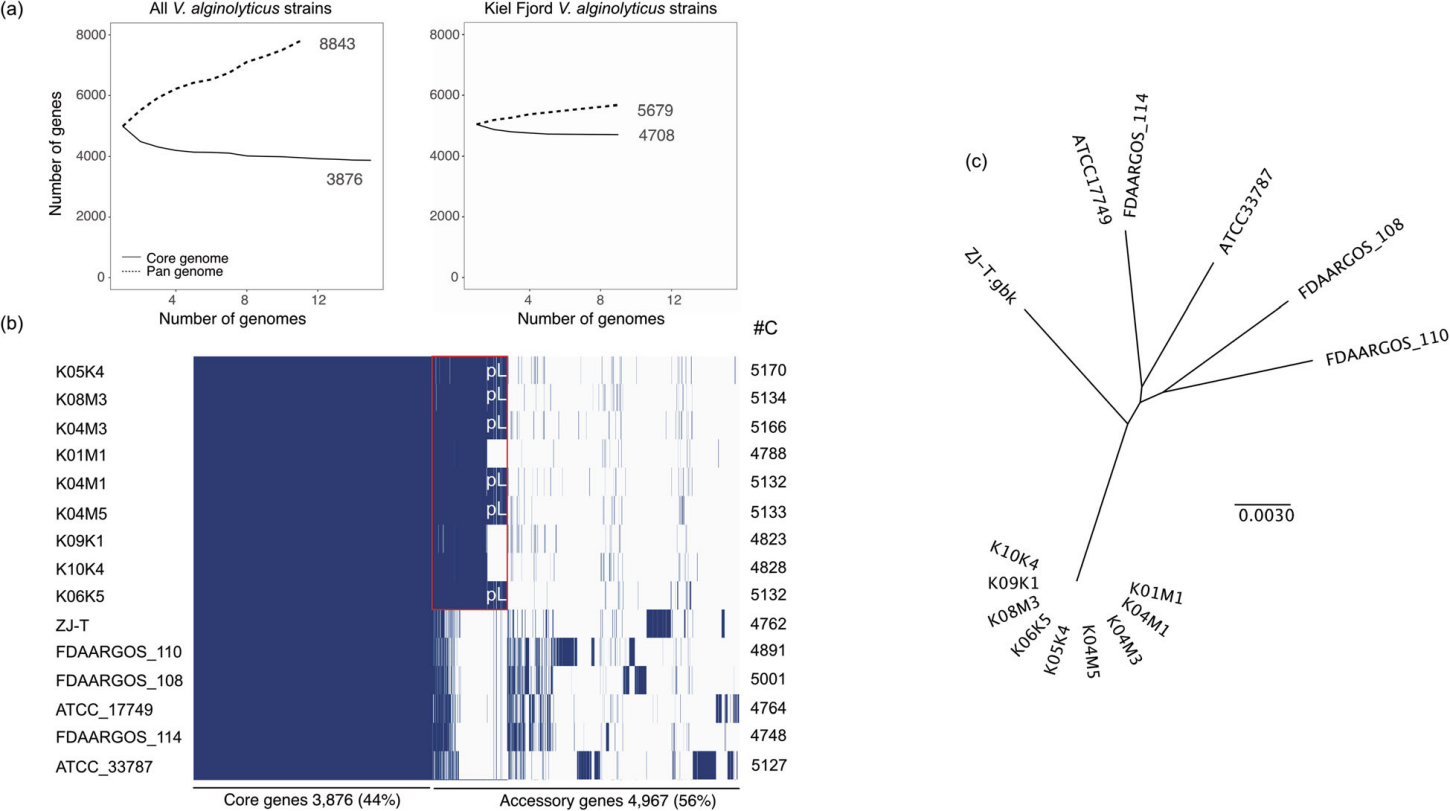
Pan-core-genome analysis of 15 closed *Vibrio alginolyticus* strains. **a** Number of gene clusters for pan (dashed line) and core genome (solid line) resulting from the sequential addition of genomes. The curve shows mean values of multiple iterations (> 100) where genomes were randomly added and the pan- and core-genome was calculated successively for all 15 steps (left: All 15 *V. alginolyticus* strains) and nine steps (right: Kiel-Fjord *V. alginolyticus* only). **b** Gene presence (dark blue) -absence (light blue) matrix showing the distribution of core and accessory genes for each of the 15 *V. alginolyticus* genomes. Each row corresponds to one strain, each column represents an orthologous gene family. Numbers on the right (#C) correspond to number of gene clusters per genome. The red square indicates the core-region specific for the Kiel isolates, the six mega-plasmids are indicated by white pL letters. **c** Bayesian phylogenetic tree of all 15 isolates based on the core-genome alignment

To compare the gene repertoire of the nine *V. alginolyticus* strains from the Kiel-Fjord with the global *V. alginolyticus* gene repertoire, we calculated their pangenome including the amount of core-and accessory genes as well as strain-specific singletons, i.e. genes unique to single strains [35]. The analysis included in total 73,277 protein-sequences encoded on chromosomes and MGEs, including plasmids and extra-chromosomal phage replicons. Overall, we found that the size of the pangenome of *V. alginolyticus* increased stronger (from 4997 to 8843 gene clusters) with the sequential addition of each new genome when we included all 15 strains from diverse habitats (Fig. 2a). In contrast, we only observed a slight increase in the pangenome (from 5044 to 5679 gene clusters) when we only included the nine *V. alginolyticus* isolates from the Kiel-Fjord (Fig. 2b). An increase in the pangenome results from new strain-specific genes, found in each newly analyzed isolate. The much larger increase in the pangenome across all available *V. alginolyticus* isolates relative to the increase in the pangenome of Kiel-Fjord isolates suggests that the genomic diversity of *V. alginolyticus* within the Kiel-Fjord is very limited. However, the species *V. alginolyticus* per se has a vast reservoir of genomic diversity.

We extrapolated both, the within-Kiel and the global *V. alginolyticus* pangenome by fitting a least-squares curve based on Heaps’ Law [1] and found that the within-Kiel pangenome is closed (α = 1.12), whereas the global *V. alginolyticus* pangenome is open (α = 0.58). This means, that within the global *V. alginolyticus* pangenome each newly sequenced *V. alginolyticus* isolate will reveal a unique set of genes, irrespective of the number of strains included in the present analysis. This open *V. alginolyticus* pangenome reflects the diversity in habitats in which this species exists. These distinct environments probably require different adaptations and/ or promote high levels of HGT. In contrast, a closed pangenome, as has been detected for the within-Kiel *V. alginolyticus* pangenome, suggests that the number of genes that will be obtained from any newly sequenced isolate will converge to zero. Here, we assume, that at least the sequenced pipefish associated *V. alginolyticus* strains within the Kiel-Fjord contain the major part of the gene equipment that is requested to adapt to their habitat. Thus the genomes can be expected to be highly similar, potentially resulting from niche adaptation and strong selection thereof. Strong positive selection of such adaptive genes might have led to a clonal expansion of the Kiel-*alginolyticus* ecotype. Indeed, we found no sequence divergence based on core-genomic signature among all nine isolates from Kiel (Fig. 2c), indicating a clonal expansion of this ecotype. It remains to be investigated, whether free-living *V. alginolyticus* strains or isolates from other eukaryotic hosts from the Kiel-Fjord share the same gene-pool or are more divergent from the pipefish-associated strains.

#### Core-genome and singletons

We observed a stronger decrease in the core-genome when we included all *V. alginolyticus* isolates as opposed to when we performed the analysis only within the Kiel-*alginolyticus* ecotype. Comparative analyses between all 15 *V. alginolyticus* strains and the Kiel-*alginolyticus* ecotype only, revealed that the core genome (4708 gene clusters) is four times larger than the accessory genome (971 gene clusters) when we only included the nine Kiel strains. However, when we extended the analysis and included all 15 *V. alginolyticus*, we found that the core genome (3876 gene clusters) becomes smaller than the accessory gene pool (4967 gene clusters). In other words, within the Kiel-Fjord, different *V. alginolyticus* isolates have a large core-genome (83% of the pangenome) with a limited accessory gene pool (17% of the pangenome). In contrast, despite all being members of the same species, the global accessory gene-pool is highly variable (56% of the pangenome) and the *V. alginolyticus* core-genome constitutes only 44% of the pangenome.

### Habitat specific chromosomal regions

**Fig. 3:**
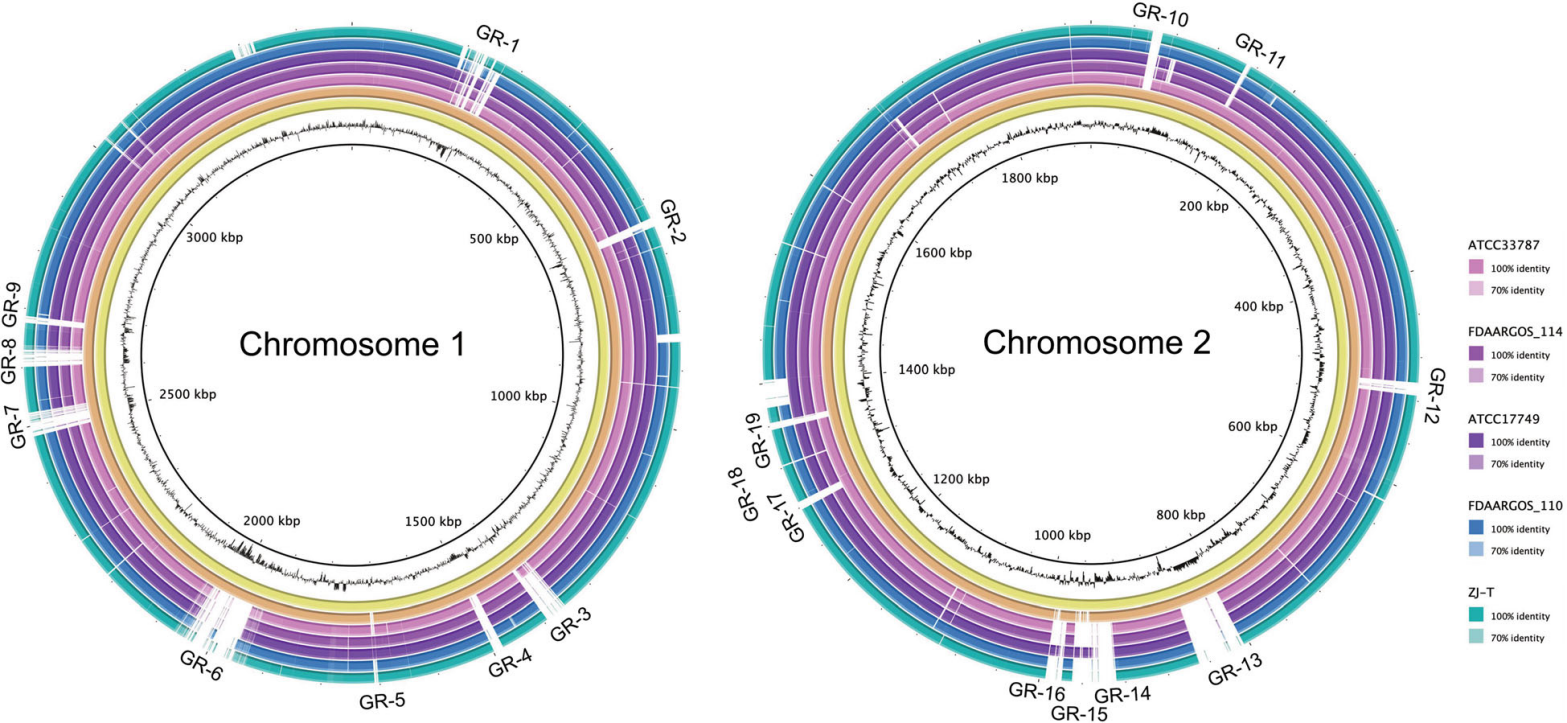
Comparison of all nine Kiel isolates and six other fully closed *V. alginolyticus* isolates. Blast matches between all strains are shown as concentric colored rings on a sliding scale according to percentage identity (100 and 70%). For chromosome 1 (left) we used K10K4 as a reference (inner black circle), as this strain has more integrated prophages than the other Kiel isolates. The second ring (yellow) corresponds to strain K05K4 which has the same integrated prophages as K10K4, the third ring (orange) corresponds to all other strains from Kiel, which miss GR 5, i.e. *Vibrio* phage VALGΦ8. For the same reasons, we used strain K04M3 as a reference for chromosome 2. Here, the second ring (yellow) corresponds to strain K04M5, which has a similar prophage composition than K04M3 while the third ring (orange) corresponds to all other Kiel strains, which miss the multiphage-cassette consisting of repeats of both filamentous *Vibrio* phages VALGΦ6 and *Vibrio* phage VALGΦ8. GC content is shown in black. Genomic Regions (GRs) unique for Kiel isolates, which have additionally been predicted using IslandViewer are annotated on the outside

The acquisition of entire gene-blocks (genomic islands, plasmids, prophages) by HGT can rapidly alter the life-style of a bacterium in quantum leaps [36]. This mechanism seems to be particularly important for bacterial adaptation to new ecological niches but also for how bacteria diverge from each other, forming ecotypes and ultimately new species [37]. Genomic islands, which encode specific functions allowing for niche adaptation and maybe even speciation events are common within the genus *Vibrio* [38]. For the Kiel-*alginolyticus* ecotype, we could identify 19 chromosomal genomic regions (GRs) of which most are unique to the strains isolated from the Kiel-Fjord (Fig. 3, Table S2). Overall, these 19 GRs encode a total of 487 genes out of which 305 have only been found within the Kiel-*alginolyticus* ecotype. Out of these 19 GRs five could be assigned to integrated prophages. GR 13 and GR 14 correspond to *Vibrio* phage VALGΦ2/2b and *Vibrio* phage VALGΦ6, which are unique for the isolates from the Kiel-Fjord and have not been found elsewhere [19]. GR 3 corresponds to *Vibrio* phage VALGΦ1, which has so far only been detected in *V. alginolyticus* ATCC33787, with a relatively low query cover of 57%, thus making it a unique region for Kiel. In contrast GR 5, which corresponds to the filamentous phage *Vibrio* phage VALGΦ8 on chromosome 1, is absent in most Kiel strains, except K10K4 and K05K4, but present in some non-Kiel strains, such as ATCC17749 and FDAARGOS_114, suggesting that GR 5 is not unique for the Kiel isolates. Similarly, GR 15, which corresponds to a multiphage-cassette consisting of multiple repeats of a combination of the two filamentous phages *Vibrio* phage VALGΦ6 and *Vibrio* phage VALGΦ8 [19], is absent in most Kiel strains, except K04M3 and K04M5 but in parts present in ATCC17749 and thus not unique for the Kiel system. Of the remaining 14 GRs, which do not correspond to integrated phages, we could identify four genomic islands: GR 2, GR 6, GR 7 and GR 8 (> 10 kb, presence of integrase/ transposase, different GC content). According to functional COG categories, these islands contain a variety of proteins, most of them involved in replication, recombination, and repair [L] and transcription [K] (see Table S3 for functional annotation of all GRs). The high prevalence of those maintenance genes is however not surprising and might result from an identification bias, as they are usually better annotated for MGEs than accessory genes, which would provide a selective advantage to the host. As such, many proteins were classified as phage integrases, transposases or other proteins, involved with viral integration into host DNA or DNA repair, suggesting that HGT might have played an important role during the acquisition of these GIs. GR 6 encodes the multi-drug transporter subunit MdtN and two other loci which were assigned to COG category [V] and predicted to be involved in defense mechanisms. This suggests that GR 6 might play a role in resistance. None of the other genomic islands could be identified as pathogenicity−/ metabolic- or resistance island. All other GRs were either smaller than 10 kb or did not contain integrases or transposases and are thus referred to as genomic regions instead of genomic island. Out of these GRs, GR 16 encoded three proteins associated with the type VI secretion system and GR 18 encoded a beta-lactamase protein suggesting an adaptive role in virulence and resistance, respectively. The acquisition of these unique GRs might have allowed the Kiel-*alginolyticus* ecotype to invade a new niche, which was then followed by clonal expansion of this ecotype. Clonal expansion has been observed for several pathogens, in particular within the genus *Vibrio*. The best characterized example of clonal expansion upon acquisition of virulence genes is *V. cholerae*. But also other *Vibrio* pathogens, for instance *V. parahaemolyticus* have been shown to experience similar evolutionary dynamics (for a review see [39]).

### Genomic differences within Kiel-alginolyticus ecotype

**Fig. 4:**
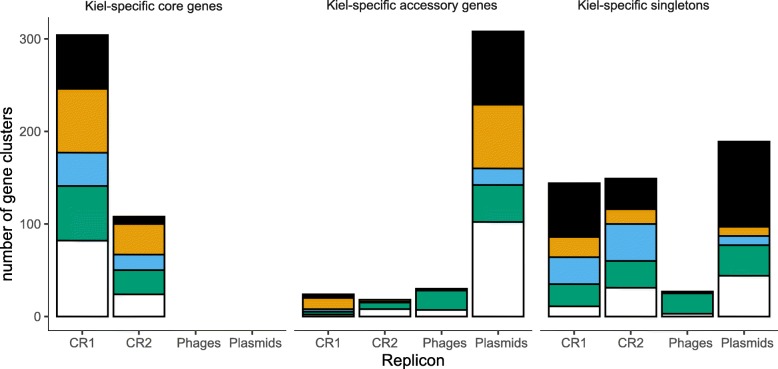
Genomic variation among all nine Kiel *V. alginolyticus* strains. Left: Kiel-specific core genes (shared by all nine isolates), middle: Kiel-specific accessory genes (shared by two or more isolates, but not by all), and right: Kiel-specific singletons (unique for one isolate) distributed across replicons (CR1: chromosome 1, CR2: chromosome 2, Phages: extrachromosomal phages, Plasmids) and assigned to Clusters of Orthologous Groups of Proteins (COGs): black: information storage and processing; orange: cellular processes and signaling; blue: metabolism; green: poorly characterized; white: not assignable

To investigate genomic differences between the nine strains from the Kiel-Fjord, we focused on all those gene-clusters from the Roary analysis, which could only be found within the Kiel-*alginolyticus* ecotype but were absent in all non-Kiel isolates. We found that all Kiel-specific core-genes (*n* = 412) were located exclusively on one of the two chromosomes (Fig. 4). In contrast, the majority of the Kiel-specific accessory gene clusters (89%) were encoded on mobile genetic elements, in particular plasmids. These results support ongoing theory that accessory genes are predominately located on MGEs and shared by horizontal gene transfer (HGT) [5]. We found 490 gene clusters with no orthologous in any other Kiel strain, i.e. singletons out of which 40% were located on MGEs, in particular, extrachromosomal replicating elements (170 on plasmids and 27 on extrachromosomal phages) and 60% (*n* = 293) were chromosomal (Fig. 4). All Kiel-specific *alginolyticus* gene clusters were further assigned to putative functional categories using the Clusters of Orthologous Groups of Proteins (COG) database [40] (Table S4). Even though a large fraction of the gene clusters could either not be assigned to a COG or was poorly characterized, we found differences in the relative distribution of super-functional COGs between core- and accessory genomes and singletons: The majority of the singletons (37%) was predicted to be dedicated to cellular processes/ signaling, while relatively small proportions of gene clusters (10 and 16%) belong to information storage/ processing and metabolism. In contrast, the largest proportion of gene-clusters encoded on core-genes was predicted to belong to information storage/ processing (24%), and only 16 and 13% of gene-clusters encoded on core genes belong to cellular processes/ signaling and metabolism. Among the gene-clusters encoded on the accessory genome 22% could be assigned to information storage/ processing as well as to cellular processes/ signaling and only 6% to the metabolism (Fig. 4). Within the accessory genome most of the genes are involved in replication, recombination, and repair (COG [L], Table S4). These include mainly proteins involved in HGT, such as transposases, integrases, transferases, recombinases as well as proteins involved in immunity, such as CRISPR associated helicase Cas3 and restriction modification methylases. This extensive representation of proteins involved in HGT on the accessory genome suggests that HGT was potentially one of the driving evolutionary mechanisms underlying the diversification of the nine *V. alginolyticus* strains from the Kiel-Fjord.

**Fig. 5:**
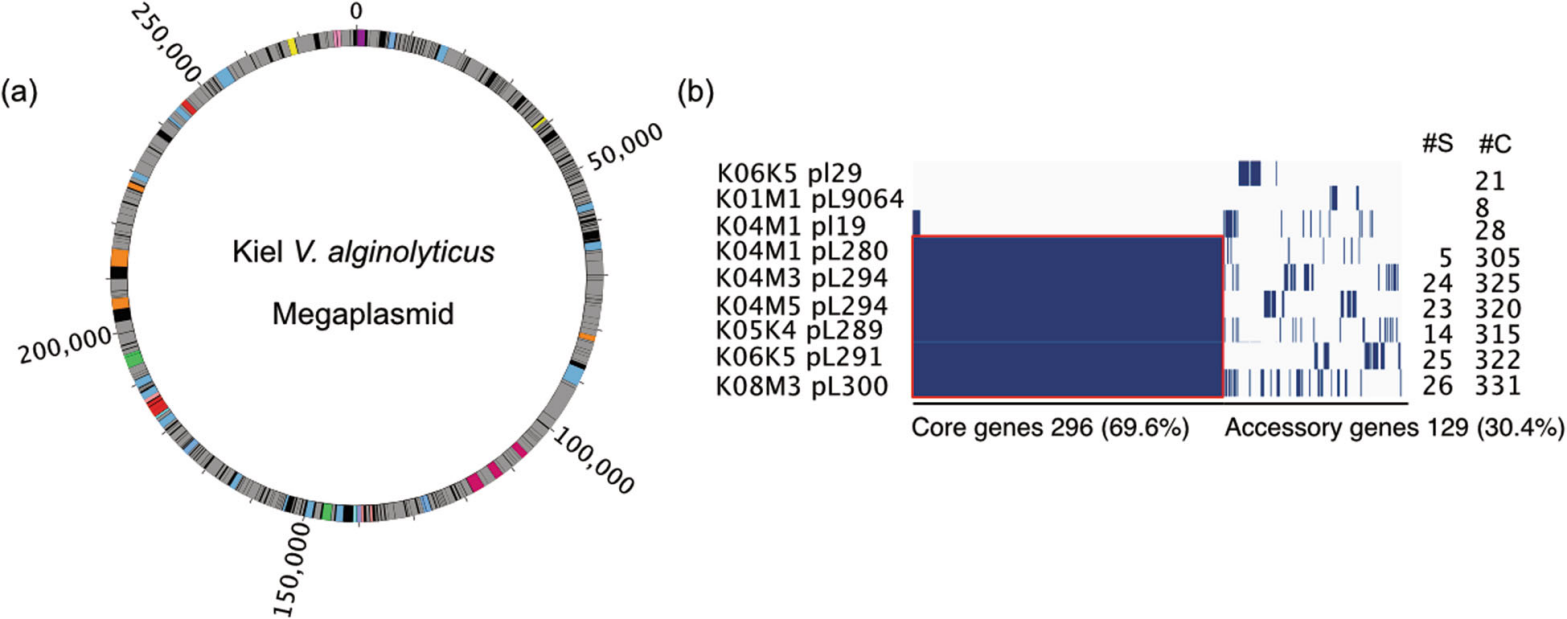
Comparative analysis of the Kiel *alginolyticus* mega plasmid. **a** Plasmid map. ORFs are color-coded according to COG functional categories: Purple: S, yellow: D, pink: EH, pink: K, blue: L, black: NA, magenta: NU, red: O, light-blue: S, green: T, orange: U, sky-blue: V, grey: hypothetical proteins. **b** Gene presence (dark blue) -absence (light blue) matrix showing the distribution of core and accessory genes for each of the six mega-plasmids. Each row corresponds to one strain, each column represents an orthologous gene family. The red square represents the core genome of the mega-plasmids (296 genes) as opposed to the number of accessory genes of the mega-plasmids (129 genes). Numbers on the right correspond to (#S) number of singletons per plasmid and (#C) number of gene clusters per plasmid

The majority of the accessory gene-pool within the Kiel-*alginolyticus* ecotype is located on plasmids (Fig. 4). We could identify four different plasmids from all nine Kiel *V. alginolyticus* isolates. Three plasmid types isolated from the Kiel strains were unique for specific strains with a size of 0.9 to 2.9 kbp (Table 1). The fourth plasmid type was characterized as a mega-plasmid (Fig. 5a), which ranged between 280 and 300 kbp in size, and was present in six out of the nine strains (Fig. 5b). The six mega-plasmids share 296 core-genes, encode 129 accessory genes and between 5 and 26 singletons per plasmid (Fig. 5b). Together with the three small plasmids, plasmid-encoded singletons make up 34.5% of all 486 Kiel-specific singletons. The majority of the plasmid-specific singletons comprise hypothetical proteins. The remaining singletons include AAA proteins (K04M1_pL280), PFAM phosphoadenosine phosphosulfate reductase and ATPases (K04M5_pL294), spore germination and type IV secretion system (K5K4_pL289), endonuclease and site-specific methyl-transferase, potentially forming a restriction-modification system (K06K5_pL291), and DNA polymerase (K08M3_pL300). *V. alginolyticus* ATCC 33787 contains as well three plasmids including plasmid pMBL287, which is similar in size as the Kiel mega plasmids [29]. However, a comparison of ATCC 33787 plasmids revealed no sequence similarity to any of the plasmids from the Kiel strains.

Only 6.5% of the 870 Kiel-specific accessory genes and singletons are located on extrachromosomal phages. The within-Kiel variation caused by these phages can be explained by the absence of extrachromosomal replicons of *Vibrio* phage VALGΦ8 in all but two strains (K04M1 and K05K4). However, four strains (K04M3, K04M5, K05K4, and K10K4) had an intra-chromosomal version of this phage, while strains K01M1, K06K5 and K08M3 did neither contain an intra- nor an extrachromosomal version of *Vibrio* phage VALGΦ8 (for a detailed analysis of Kiel vibriophages see [19]). Parts of *Vibrio* phage VALGΦ8 could be identified on both chromosomes of ATCC 17749^T^, and on chromosome 1 of FDAARGOS_114 but not on any other non-Kiel *Vibrio*. Genome analyses of *Vibrio* phage VALGΦ8 revealed no significant virulence-associated genes nor any other genes that could be associated with a habitat-specific adaptation [19].

### Virulence and resistance of the Kiel-Fjord V. alginolyticus isolates

**Fig. 6:**
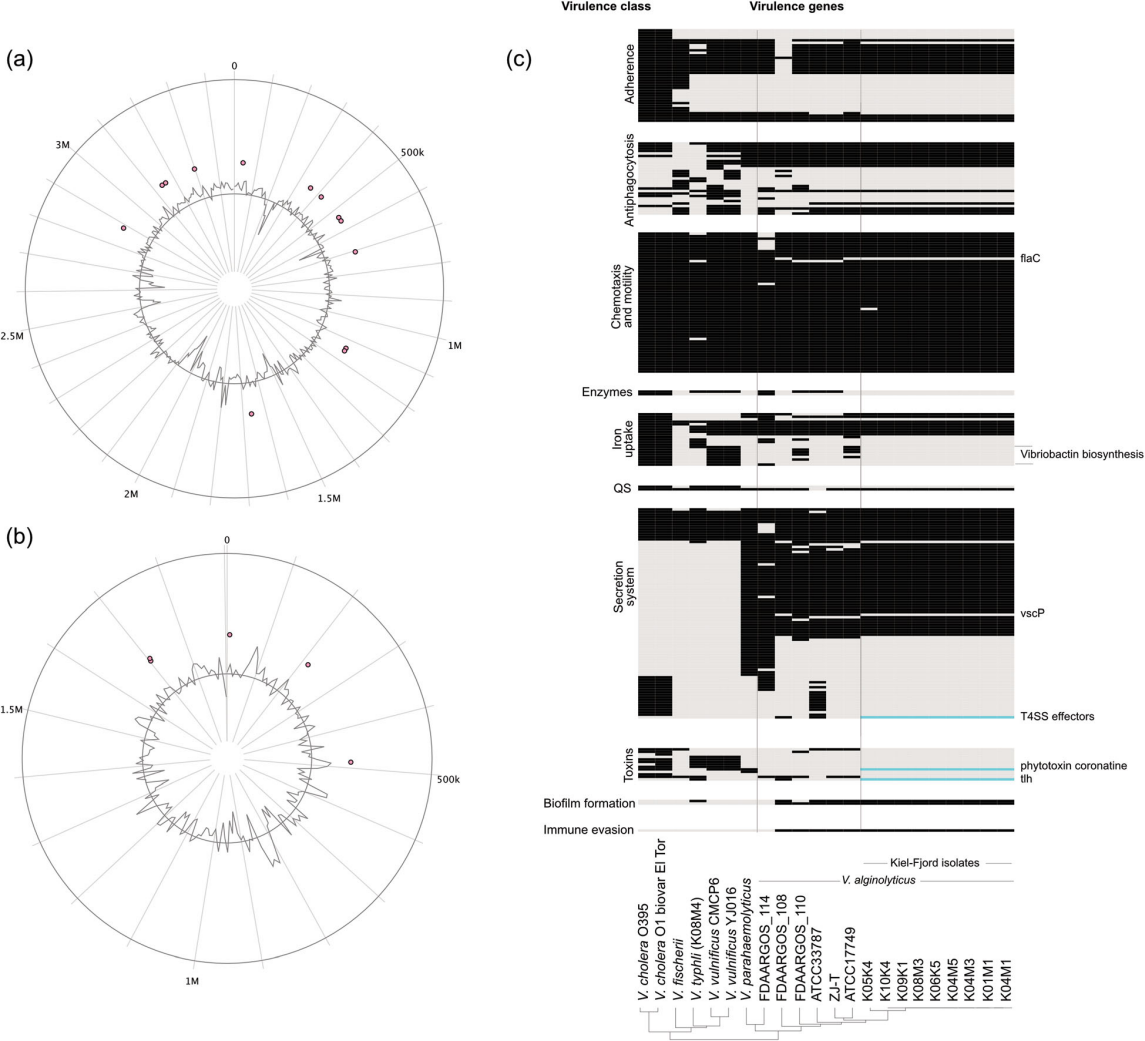
Resistance and virulence genes. Resistance genes are shown for (**a**) chromosome 1 and (**b**) chromosome 2. (**c**) Virulence genes, presented as a presence (black) / absence (grey) matrix, are grouped into 10 different virulence classes (left). Those virulence genes, which contribute to the unique profile of the Kiel-*alginolyticus* ecotype are labeled on the right and if present, marked in turquoise. Hierarchical cluster analysis of the 22 strains (bottom), which were incorporated into this analysis shows that the Kiel-*alginolyticus* ecotype can be separated from all other *V. alginolyticus* strains solely based on presence/ absence of specific virulence genes

We found an identical virulence and resistance profile among the nine *V. alginolyticus* isolates from the Kiel-Fjord. The Kiel-Fjord isolates encode a total number of 17 homologues resistance genes out of which the majority (*n* = 13) is located on chromosome 1 and four resistance genes are encoded on chromosome 2 (Fig. 6, Table S5). In comparison with other *V. alginolyticus* strains (~ 25–130 resistance genes on Chromosome 1 and 11–12 resistance genes on Chromosome 2), isolates from the Kiel-Fjord have significantly less resistance genes (t-test: t_8.85_ = 3.51, *p* = 0.007; Fig. 6) and are missing in particular genes conferring resistance to tetracycline, aminoglycosides, and quinolones.

In contrast to non-pathogenic strains, such as *V. fischerii*, which is a mutualist containing 112 virulence genes, the Kiel-Fjord isolates encode more virulence genes (150 virulence genes, exception strain K05K4, 149 virulence genes). This number is lower than what has been found for human pathogenic v*ibrios*, such as *V. cholerae* (~ 165 virulence genes) and *V. parahaemolyticus* (162 virulence genes), but similar to what has been found in other *V. alginolyticus* strains, except (strain FDAARGOS_114: 165 virulence genes). Unique for the Kiel-Fjord isolates is the absence of genes involved in Vibriobactin biosynthesis, which are present in almost all other *V. alginolyticus* strains. Similarly, In contrast to other *V. alginolyticus* isolates, the strains from the Kiel-Fjord miss the gene flaC, which is involved in the regulation of stringent-response-induced toxin production [41] and vscP, a gene involved in the type III secretion system. In contrast to other *V. alginolyticus* strains, the Kiel-*alginolyticus* ecotype encodes the type IV secretion system effectors, the phytotoxin coronatine and the thermolabile hemolysin (tlh), both toxins, which could not be found in any other *V. alginolyticus* strain. This unique virulence profile of the Kiel-Fjord isolates separates them from other *Vibrio* species including other *V. alginolyticus* isolates abut also another strain from the Kiel-Fjord: *V. typhli*, K08M4 (Chibani C, Hertel R, Goehlich H, Mertens V, Bunk B, Overmann J, et al. Complete Genome Sequence of Vibrio typhli K08M4, a fish pathogen isolated from the Kiel Fjord. in preparation) (Fig. 6). This clear separation was further supported by a hierarchical cluster analysis (Fig. 6), which indicates, that only based on the presence/absence of virulence genes the Kiel-*alginolyticus* ecotype can be distinguished from all other *V. alginolyticus* strains, sequenced to date. The unique resistance and virulence profile within the Kiel-Fjord *V. alginolyticus* isolates might be a further indication for niche-adaptation followed by clonal expansion of this ecotype.

## Conclusions

By using a multi-scale comparative genomic approach, we conclude that the nine *V. alginolyticus* strains isolated from pipefish in the Kiel-Fjord belong to a unique ecotype, which evolved from a clonal expansion following niche adaptation. We came to this conclusion based on the following observations. First, the nine strains from the Kiel-Fjord have a closed-pangenome reflecting low genomic diversity within its niche. Second, even though these strains have been isolated from different eukaryotic host species, they did not differ based on core-genomic signatures which has been previously suggested based on MLSA [23]. Third, niche adaptation and ultimately speciation in the genus *Vibrio* have been suggested to be mainly driven by HGT [13]. Likewise, unique GRs and a unique repertoire of MGEs within the Kiel-Fjord isolates (including unique plasmids and prophages) support ongoing theory that the acquisition of gene-blocks by HGT, one of the most important mechanisms driving the evolution of niche adaptation and clonal expansion also played an important role in the evolution of the Kiel-*alginolyticus* ecotype. Lastly, the unique virulence and resistance profile which clearly separates the Kiel-Fjord isolates from all other investigated *V. alginolyticus* strains, suggests, that these are essential genes, required for a successful colonization of the pipefish, the niche of the Kiel-*alginolyticus* ecotype. To ultimately verify this theory, colonization experiments with knock-out mutants lacking the Kiel-specific virulence genes, or alternatively non virulent *Vibrio* strains equipped with the Kiel-specific virulence genes, would be required.

We are aware that our conclusion is limited by the low number of non-Kiel *V. alginolyticus* strains available included in the comparative genomic analyses. We could have included draft genomes, to increase the number of sequences available for comparison. However, due to the incomplete information especially for MGEs stored in draft genomes, the number of core-genomes drops considerably by approximately 50% compared to when only complete genomes are included in the analysis [20]. In favor of a more accurate analysis, we thus decided to only include complete genomes.

The present study extends our current knowledge of pan-and core-genomic signatures in the genus *Vibrio* which is currently heavily biased towards human pathogens, such as *V. cholerae* and *V. parahaemolyticus*. Based on the present results we suggest that also non-human pathogens might experience similar evolutionary dynamics, once relevant pathogenic traits (in the present case: *Vibrio* phage VALGΦ6 [19], plus three unique virulence genes (Fig. 6)) have been acquired. The availability of a suitable niche, in the present case, presumably the pipefish, might have led to niche adaptation and clonal expansion of the Kiel-*alginolyticus* ecotype, clearly separating it from other *V. alginolyticus* species.

## Methods

### Bacterial genome data

Using a combination of PacBio and Illumina sequencing, we generated eight closed genomes and one permanent draft genome of nine *Vibrio* strains isolated from different pipefish in the Kiel-Fjord [28] and previously characterized as *V. alginolyticus* based on multi-locus-sequence typing [23]. For sequencing and annotation information please see [23]. We additionally sequenced prophages induced with mitomycin C from each strain, for details see [19]. We compared all replicons from the nine *V. alginolyticus* strains to 10 closed *Vibrio* genome sequences downloaded from the NCBI nucleotide database; date of accession 1.12.2019 (Table S1).

### Comparative genome analysis

#### Average nucleotide identity

The average similarity between the nine *V. alginolyticus* genomes from the present study, six other complete *V. alginolyticus* genomes as well as four other closely related *vibrios* (i.e. three *V. diabolicus* and one *V. antiquarius*) was measured as the average nucleotide identity (ANI) of all 19 *Vibrio* replicons (concatenated Chromosome 1 and Chromosome 2) using the ANIm mode from the PyANI python module [42] based on MUMmer (https://github.com/widdowquinn/pyani). Briefly, nucleotide sequences were extracted from each GenBank file using Biopython (https://biopython.org/) and subsequently used as input for PyANI for genome sequence alignment.

#### Pan-Core genome prediction

To determine the total gene-pool of *V. alginolyticus* within the Kiel-Fjord as well as the global gene repertoire of *V. alginolyticus,* we performed two pan-core genome analyses. In the first analysis, we only included the nine strains from the Kiel-Fjord. In the second analysis, we included all fully closed *V. alginolyticus* genomes available from NCBI (accession date 10.10. 2019) which were isolated from various regions (Table 1), in addition to the nine Kiel strains. Genbank files of all closed *V. alginolyticus* isolates were converted to GFF3 files and a nucleotide alignment was generated using Roary 3.8.0 [43] with standard settings (minimum BLASTP identity of 90% with paralogue splitting). We used concatenated files for each isolate including both chromosomes and if existing, mobile genetic elements (plasmids and episomal phages). To estimate whether we have an open or a closed pangenome we used Heaps’ Law: *η* = *κ* ∗ *N*^−*α*^ [1], implemented in the R package ‘micropan’ [44], where η is the expected number of genes for a given number of Genomes (N), and κ and α are constants to fit the specific curve. The exponent α is an indicator whether the pangenome is open (α < 1) or closed (α > 1). Lastly, a functional characterization of all protein-coding sequences was performed using the eggnog-mapper v2 database v5.0 [45, 46]. We subsequently determined the COG category (Cluster of Orthologous Groups of Proteins [40]) for Kiel-specific core- and accessory genes as well as singletons identified by the pan-core analysis based on the Roary clustering input table.

#### Phylogenetic analysis

To further infer the phylogenetic relationship among all *V. alginolyticus* isolates we generated a phylogenetic tree based on the core-genome alignment for all 15 strains obtained from the Roary analysis (length for all strains ~ 3.84 Mbp, which corresponds to approximately 72% of the average size of the input genomes). To do so, we used the Bayesian Markov chain Monte Carlo (MCMC) method as implemented in MrBayes version 3.2.5 [47, 48]. The TN93 [49] model plus invariant sites (TN93 + I), as suggested by the Akaike information criterion (AIC) given by jModelTest [50], was used as a statistical model for nucleotide substitution. The MCMC process was repeated for 10^6^ generations and sampled every 5000 generations. The first 2000 trees were deleted as burn-in processes and the consensus tree was constructed from the remaining trees. Convergence was assured via the standard deviation of split frequencies (< 0.01) and the potential scale reduction factor (PSRF~ 1). The resulting phylogenetic tree and associated posterior probabilities were illustrated using FigTree version 1.4.2 (http://tree.bio.ed.ac.uk/software/figtree/).

#### Genomic Islands

We used IslandViewer [51] to predict putative genomic islands of all nine isolates from the Kiel-Fjord. The selection criterion for genomic islands was based on the presence of mobile-related genes (integrases or transposases), a size > 8 kb, and a different GC content compared to the rest of the genome. To visualize the genomic islands, we used BLAST Ring Image Generator [52, 53] and created a circular map per chromosome of all fully closed *V. alginolyticus* genomes. Functional characterization of all protein-coding sequences found on the putative genomic islands was performed as described above using the eggNOG-mapper v2 database v5.0 [45, 46] where the resulting COG assignments were used for further analysis.

#### Plasmids

We used Easyfig [54] for pairwise plasmid sequence comparisons and synteny comparisons with an *E*-value cut-off of 1e− 10. Plasmid maps were generated using SnapGene Viewer (version 4.3.10) with annotated genbank files as input files. The output resulting from Roary was used to determine orthologous genes, while the output of eggNOG-mapper [45, 46] was used to assign a functional characterization to the coding protein sequences of the six mega-plasmids detected in the Kiel-specific *V. alginolyticus* isolates.

#### Virulence and resistance

The virulence of the nine Kiel-isolates was determined using controlled infection experiments in a previous study [19], and could in part be explained by the presence and the coverage of a virulence-gene carrying filamentous phage *Vibrio phage* VALGΦ6. To determine other potential chromosomal encoded virulence factors, we scanned all 15 *V. alginolyticus strains* and seven other strains from different *Vibrio* clades using the Virulence Factor Database (https://www.mgc.ac.cn/VFs/main.htm) [55]. We used IslandViewer [51] to identify homologues of resistance genes in all 15 *V. alginolyticus* strains.

All statistics and visualization graphs were performed using the ggplot2 library [56] in R 3.1.2 unless otherwise stated.

## Supplementary information

**Additional file 1: Table S1.***Vibrio* genomes accessed from NCBI 3.12.2019 used for PyANI analysis.

**Additional file 2: Table S2.** Genomic regions (GRs) unique for the nine *V. alginolyticus* strains from the Kiel-Fjord. For chromosome 1 (left) we used K10K4 as a reference, as this strain has more integrated prophages than the other Kiel isolates. For the same reason, we used strain K04M3 as a reference for chromosome 2. For a detailed comparison of GRs among all nine strains, see Fig. 3 main manuscript.

**Additional file 3: Table S3.** Functional characterization of all protein-coding sequences found on the Genomic Regions (using the eggNOG-mapper v2 software) with the resulting COG assignments. Similar to Table S2 we used strain K10K4 as reference for chromosome 1 and strain K04M3 as reference for chromosome 2. The column Kiel Specific indicates whether the GR is unique for the Kiel *alginolyticus* ecotype.

**Additional file 4: Table S4.** Functional annotation of Kiel specific gene clusters (core genome [C], accessory genes [A], singletons [S], i.e. gene cluster which was only found within the Kiel *alginolyticus* ecotype with the resulting COG assignments.

**Additional file 5: Table S5.** Resistance genes found using IslandViewer for all nine *V. alginolyticus* strains from the Kiel-Fjord. Even though this table shows the locus tags for strain K04M3 only, all other strains have been found to contain the exact same resistance genes.

## Data Availability

The genomic sequences of the nine Kiel *V. alginolyticus* strains analyzed during the current study are available at GenBank (CP017889-CP017919).

## Abbreviations

GIs: Genomic islands
GRs: Genomic regions
ANI: Average nucleotide identity
MLSA: Multi-locus-sequencing-approach
COG: Clusters of Orthologous Groups of Proteins
MGEs: Mobile genetic elements
HGT: Horizontal gene transfer

## References

[1] Tettelin H, Riley D, Cattuto C, Medini D (2008). Comparative genomics: the bacterial pan-genome. Curr Opin Microbiol.

[2] Rasko DA, Rosovitz MJ, Myers GSA, Mongodin EF, Fricke WF, Gajer P (2008). The pangenome structure of Escherichia coli: comparative genomic analysis of E-coli commensal and pathogenic isolates. J Bacteriol.

[3] Aagesen AM, Phuvasate S, Su YC, Hase CC (2013). Persistence of *Vibrio parahaemolyticus* in the Pacific oyster, *Crassostrea gigas*, is a multifactorial process involving Pili and flagella but not type III secretion systems or phase variation. Appl Environ Microbiol.

[4] Austin B, Austin DA, Blanch AR, Cerda M, Grimont F, Grimont PAD (1997). A comparison of methods for the typing of fish-pathogenic *Vibrio* spp. Syst Appl Microbiol.

[5] Medini D, Donati C, Tettelin H, Masignani V, Rappuoli R (2005). The microbial pan-genome. Curr Opin Genet Dev.

[6] Hazen TH, Pan L, Gu JD, Sobecky PA (2010). The contribution of mobile genetic elements to the evolution and ecology of *Vibrios*. FEMS Microbiol Ecol.

[7] McInerney JO, McNally A, O'Connell MJ. Why prokaryotes have pangenomes. Nat Microbiol. 2017;2(4):17040. 10.1038/nmicrobiol.2017.40. 10.1038/nmicrobiol.2017.40 28350002

[8] Brockhurst MA, Harrison E, Hall JPJ, Richards T, McNally A, MacLean C (2019). The ecology and evolution of Pangenomes. Curr Biol.

[9] Cohan FM. Transmission in the Origins of Bacterial Diversity, From Ecotypes to Phyla. Microbiol Spectr. 2017;5(5). 10.1128/microbiolspec.MTBP-0014-2016. 10.1128/microbiolspec.mtbp-0014-2016 PMC11687548 29027519

[10] Jones JL. Chapter 11 - Vibrio. In: Christine E.R. Dodd, Tim Aldsworth, Richard A. Stein, Dean O. Cliver, Hans P. Riemann, editors. Foddborne Dieseases. 3rd ed: Academic Press; 2017. p. 535–46. ISBN 9780123850072. 10.1016/B978-0-12-385007-2.18001-5.

[11] Thompson CC, Vicente ACP, Souza RC, Vasconcelos ATR, Vesth T, Alves N, et al. Genomic taxonomy of vibrios. BMC Evol Biol. 2009;9:258. Published 2009 Oct 27. 10.1186/1471-2148-9-258. 10.1186/1471-2148-9-258 PMC2777879 19860885

[12] Thompson FL, Iida T, Swings J (2004). Biodiversity of *Vibrios*. Microbiol Mol Biol R.

[13] Hunt DE, David LA, Gevers D, Preheim SP, Alm EJ, Polz MF (2008). Resource partitioning and sympatric differentiation among closely related bacterioplankton. Science..

[14] Banks DJ, Beres SB, Musser JM (2002). The fundamental contribution of phages to GAS evolution, genome diversification and strain emergence. Trends Microbiol.

[15] Ohnishi M, Kurokawa K, Hayashi T (2001). Diversification of Escherichia coli genomes: are bacteriophages the major contributors?. Trends Microbiol.

[16] Castillo D, Kauffman K, Hussain F, Kalatzis P, Rorbo N, Polz MF, et al. Widespread distribution of prophage-encoded virulence factors in marine Vibrio communities. Sci Rep. 2018;8(1):9973. Published 2018 Jul 2. 10.1038/s41598-018-28326-9. 10.1038/s41598-018-28326-9 PMC6028584 29967440

[17] Di Lorenzo M, Stork M, Tolmasky ME, Actis LA, Farrell D, Welch TJ (2003). Complete sequence of virulence plasmid pJM1 from the marine fish pathogen Vibrio anguillarum strain 775. J Bacteriol.

[18] Dunn AK, Martin MO, Stabb EV (2005). Characterization of pES213, a small mobilizable plasmid from Vibrio fischeri. Plasmid..

[19] Chibani CM, Hertel R, Hoppert M, Liesegang H, Wendling CC. Closely related Vibrio alginolyticus strains encode an identical repertoire of prophages and filamentous phages. bioRxiv. 2019:859181. 10.3390/v12121359 PMC7761403 33261037

[20] Lukjancenko O, Ussery DW. Vibrio chromosome-specific families. Front Microbiol. 2014;5:73. 10.3389/fmicb.2014.00073. 10.3389/fmicb.2014.00073 PMC3957060 24672511

[21] Lee KK, Yu SR, Yang TI, Liu PC, Chen FR (1996). Isolation and characterization of Vibrio alginolyticus isolated from diseased kuruma prawn, *Penaeus japonicus*. Lett Appl Microbiol.

[22] Gonzalez-Escalona N, Blackstone GM, DePaola A (2006). Characterization of a Vibrio alginolyticus strain, isolated from Alaskan oysters, carrying a hemolysin gene similar to the thermostable direct hemolysin-related hemolysin gene (trh) of Vibrio parahaemolyticus. Appl Environ Microbiol.

[23] Wendling CC, Piecyk A, Refardt D, Chibani C, Hertel R, Liesegang H (2017). Tripartite species interaction: eukaryotic hosts suffer more from phage susceptible than from phage resistant bacteria. BMC Evol Biol.

[24] Wendling CC, Wegner KM. Relative contribution of reproductive investment, thermal stress and Vibrio infection to summer mortality phenomena in Pacific oysters. Aquaculture. 2013;412–3:88–96.

[25] Poirier M, Listmann L, Roth O (2017). Selection by higher-order effects of salinity and bacteria on early life-stages of Western Baltic spring-spawning herring. Evol Appl.

[26] Le Roux F, Wegner K, Austin C, Vezzulli L, Osorio C, Amaro C (2015). The Emergence of Vibrio pathogens in Europe: Ecology, Evolution and Pathogenesis (Paris, 11–12 March 2015). Front Microbiol.

[27] Wendling CC, Fabritzek AG, Wegner KM. Population-specific genotype x genotype x environment interactions in bacterial disease of early life stages of Pacific oyster larvae. Evol Appl. 2017;10(4):338–47. Published 2017 Mar 9. 10.1111/eva.12452. 10.1111/eva.12452 PMC5367073 28352294

[28] Roth O, Keller I, Landis SH, Salzburger W, Reusch TB (2012). Hosts are ahead in a marine host-parasite coevolutionary arms race: innate immune system adaptation in pipefish *Syngnathus typhle* against *Vibrio phylotypes*. Evolution..

[29] Wang P, Wen Z, Li B, Zeng Z, Wang X (2016). Complete genome sequence of Vibrio alginolyticus ATCC 33787(T) isolated from seawater with three native megaplasmids. Mar Genomics.

[30] Deng Y, Chen C, Zhao Z, Huang X, Yang Y, Ding X. Complete Genome Sequence of Vibrio alginolyticus ZJ-T. Genome Announc. 2016;4(5):e00912–16. 10.1128/genomeA.00912-16. 10.1128/genomeA.00912-16 PMC5009981 27587824

[31] Liu X-F, Cao Y, Zhang H-L, Chen Y-J, Hu C-J. Complete genome sequence of Vibrio alginolyticus ATCC 17749T. Genome Announc. 2015;3(1):e01500–14. 10.1128/genomeA.01500-14. 10.1128/genomeA.01500-14 PMC4319515 25635021

[32] Okada K, Iida T, Kita-Tsukamoto K, Honda T (2005). Vibrios commonly possess two chromosomes. J Bacteriol.

[33] Goris J, Konstantinidis KT, Klappenbach JA, Coenye T, Vandamme P, Tiedje JM (2007). DNA-DNA hybridization values and their relationship to whole-genome sequence similarities. Int J Syst Evol Microbiol.

[34] Yoon SH, Ha SM, Lim J, Kwon S, Chun J (2017). A large-scale evaluation of algorithms to calculate average nucleotide identity. Antonie Van Leeuwenhoek.

[35] Land M, Hauser L, Jun SR, Nookaew I, Leuze MR, Ahn TH (2015). Insights from 20 years of bacterial genome sequencing. Funct Integr Genomics.

[36] Groisman EA, Ochman H (1996). Pathogenicity islands: bacterial evolution in quantum leaps. Cell..

[37] Schmidt H, Hensel M (2004). Pathogenicity islands in bacterial pathogenesis. Clin Microbiol Rev.

[38] Vesth T, Wassenaar TM, Hallin PF, Snipen L, Lagesen K, Ussery DW (2010). On the origins of a Vibrio species. Microb Ecol.

[39] Shapiro BJ (2016). How clonal are bacteria over time?. Curr Opin Microbiol.

[40] Galperin MY, Makarova KS, Wolf YI, Koonin EV (2015). Expanded microbial genome coverage and improved protein family annotation in the COG database. Nucleic Acids Res.

[41] Kim HY, Yu SM, Jeong SC, Yoon SS, Oh YT (2018). Effects of flaC mutation on stringent response-mediated bacterial growth, toxin production, and motility in Vibrio cholerae. J Microbiol Biotechnol.

[42] Pritchard L, Glover RH, Humphris S, Elphinstone JG, Toth IK (2016). Genomics and taxonomy in diagnostics for food security: soft-rotting enterobacterial plant pathogens. Anal Methods-Uk.

[43] Page AJ, Cummins CA, Hunt M, Wong VK, Reuter S, Holden MT (2015). Roary: rapid large-scale prokaryote pan genome analysis. Bioinformatics..

[44] Snipen L, Liland KH (2015). micropan: an R-package for microbial pan-genomics. Bmc Bioinformatics.

[45] Huerta-Cepas J, Szklarczyk D, Forslund K, Cook H, Heller D, Walter MC (2016). eggNOG 4.5: a hierarchical orthology framework with improved functional annotations for eukaryotic, prokaryotic and viral sequences. Nucleic Acids Res.

[46] Huerta-Cepas J, Szklarczyk D, Heller D, Hernandez-Plaza A, Forslund SK, Cook H (2019). eggNOG 5.0: a hierarchical, functionally and phylogenetically annotated orthology resource based on 5090 organisms and 2502 viruses. Nucleic Acids Res.

[47] Ronquist F, Teslenko M, van der Mark P, Ayres DL, Darling A, Hohna S (2012). MrBayes 3.2: efficient Bayesian phylogenetic inference and model choice across a large model space. Syst Biol.

[48] Huelsenbeck JP, Ronquist F (2001). MRBAYES: Bayesian inference of phylogenetic trees. Bioinformatics..

[49] Tamura K, Nei M (1993). Estimation of the number of nucleotide substitutions in the control region of mitochondrial-DNA in humans and chimpanzees. Mol Biol Evol.

[50] Posada D, Buckley TR (2004). Model selection and model averaging in phylogenetics: advantages of akaike information criterion and bayesian approaches over likelihood ratio tests. Syst Biol.

[51] Dhillon BK, Chiu TA, Laird MR, Langille MG, Brinkman FS (2013). IslandViewer update: Improved genomic island discovery and visualization. Nucleic Acids Res.

[52] Méhats C, Tanguy G, Paris B, Robert B, Pernin N, Ferré F (2000). Pregnancy induces a modulation of the cAMP phosphodiesterase 4-conformers ratio in human myometrium: consequences for the utero-relaxant effect of PDE4-selective inhibitors. J Pharmacol Exp Ther.

[53] Alikhan NF, Petty NK, Ben Zakour NL, Beatson SA (2011). BLAST ring image generator (BRIG): simple prokaryote genome comparisons. BMC Genomics.

[54] Sullivan MJ, Petty NK, Beatson SA (2011). Easyfig: a genome comparison visualizer. Bioinformatics..

[55] Chen L, Yang J, Yu J, Yao Z, Sun L, Shen Y (2005). VFDB: a reference database for bacterial virulence factors. Nucleic Acids Res.

[56] Wickham H (2011). ggplot2. Wires Comput Stat.

